# The MET Oncogene: Thirty Years of Insights into Molecular Mechanisms Driving Malignancy

**DOI:** 10.3390/ph17040448

**Published:** 2024-03-30

**Authors:** Tiziana Crepaldi, Simona Gallo, Paolo Maria Comoglio

**Affiliations:** 1 Department of Oncology, University of Turin, Regione Gonzole 10, 10143 Orbassano, Italy; tiziana.crepaldi@unito.it (T.C.); simona.gallo@unito.it (S.G.); 2 Candiolo Cancer Institute, FPO-IRCCS, SP142, Km 3.95, 10060 Candiolo, Italy; 3 IFOM ETS—The AIRC Institute of Molecular Oncology, Via Adamello 16, 20139 Milano, Italy

**Keywords:** MET, HGF, genetic alterations, protein overexpression

## Abstract

The discovery and subsequent research on the MET oncogene’s role in cancer onset and progression have illuminated crucial insights into the molecular mechanisms driving malignancy. The identification of MET as the hepatocyte growth factor (HGF) receptor has paved the path for characterizing the MET tyrosine kinase activation mechanism and its downstream signaling cascade. Over the past thirty years, research has established the importance of HGF/MET signaling in normal cellular processes, such as cell dissociation, migration, proliferation, and cell survival. Notably, genetic alterations that lead to the continuous activation of MET, known as constitutive activation, have been identified as oncogenic drivers in various cancers. The genetic lesions affecting MET, such as exon skipping, gene amplification, and gene rearrangements, provide valuable targets for therapeutic intervention. Moreover, the implications of MET as a resistance mechanism to targeted therapies emphasize the need for combination treatments that include MET inhibitors. The intriguing “flare effect” phenomenon, wherein MET inhibition can lead to post-treatment increases in cancer cell proliferation, underscores the dynamic nature of cancer therapeutics. In human tumors, increased protein expression often occurs without gene amplification. Various mechanisms may cause an overexpression: transcriptional upregulation induced by other oncogenes; environmental factors (such as hypoxia or radiation); or substances produced by the reactive stroma, such as inflammatory cytokines, pro-angiogenic factors, and even HGF itself. In conclusion, the journey to understanding MET’s involvement in cancer onset and progression over the past three decades has not only deepened our knowledge, but has also paved the way for innovative therapeutic strategies. Selective pharmacological inactivation of MET stands as a promising avenue for achieving cancer remission, particularly in cases where MET alterations are the primary drivers of malignancy.

## 1. Introduction

Several laboratories have been engaged for about thirty years in the detailed study of an oncogene, identified with the acronym “MET”, that is capable of inducing and supporting the uncontrolled growth of cancer cells and—above all—the invasive and metastatic phenotype [1,2]. In 1984, Cooper and colleagues identified an oncogene in a human osteosarcoma cell line that had been induced chemically. They proposed the name “MET” for this oncogene, drawing inspiration from the mutagenic compound used in their study, N-methyl-N’-nitro-N-nitrosoguanidine [3]. The active oncoprotein was actually a fusion of two different loci from separate chromosomes [4]. This genetic alteration involved a segment from chromosome 1 at the 5′ end, known as TPR (translocated promoter region), and a part of the MET proto-oncogene from chromosome 7 at the 3′ end. This combination ensued in the expression of a chimeric mRNA, which resulted in the translation of a truncated cytoplasmic protein sharing similarities with tyrosine kinase families. The chimeric protein exhibited constitutive activation because of the spontaneous dimerization enabled by the leucine zipper domain of TPR. The MET-encoded protein was found to be a novel transmembrane tyrosine kinase featuring a dimeric structure of covalently linked alpha and beta chains [5]. Subsequently, it was proven that MET is the receptor tyrosine kinase (RTK) for HGF (hepatocyte growth factor), a cytokine associated with hepatocyte regeneration [6,7]. HGF was recognized to be identical to SF (scatter factor), a factor of cell motility [8,9]. The characterization of MET signaling commenced in 1994, revealing that MET undergoes dimerization and autophosphorylation at tyrosine residues Y1234 and Y1235 within its catalytic domain upon HGF stimulation [10]. Subsequently, the tyrosine residues 1349 and 1356 of the carboxy-terminal tail become phosphorylated, forming a tandem docking site that can attract a variety of SH2-containing signal transducers, such as Grb2 (growth factor receptor-bound protein 2), PI3K (phosphoinositide 3-kinase), PLCγ (phospholipase Cγ), and Src [10]. The GAB1 protein interacts with the activated MET receptor either directly [11] or indirectly via Grb2 [12], amplifying the MET signaling platform by providing additional docking sites for the attachment of downstream adaptor proteins. Using specific MET mutants that selectively activate either the Ras or PI3K pathways, research conducted by us and others has demonstrated that activating Ras is both essential and sufficient for cell proliferation, while targeting PI3K specifically enhances cell motility [13,14]. Further, using peptide inhibitors and dominant negative techniques, it has been found that activating STAT3 (signal transducer and activator of transcription 3) is necessary for cell polarization and the development of complex, branched tubular structures [15,16]. This indicates that it is possible to experimentally separate the complex processes involved in invasive growth and pinpoint the key players responsible for each process. The combined activation of the Ras and PI3K pathways, which stimulate cell growth and motility while inhibiting cell death, respectively, results in effective cell-cell dissociation, invasion into the extracellular matrix (ECM), and metastasis [17,18]. The oncogenic activity of MET results from the alteration of the proto-oncogene, which is present under normal conditions in all healthy organisms, whose functions have been usurped by malignant cells. The definitive link between abnormal MET activation and cancer was confirmed in 1997 through the discovery of MET mutations associated with inherited forms of renal carcinoma [19]. Pathologically, dysregulated MET activity is implicated in a wide range of cancers, such as renal [20], lung, liver, and gastric carcinomas, among others [1,2]. The risk of cancer associated with the MET gene emerges when there is aberrant activation of its signaling pathways. Mutations, rearrangements, or amplifications of the MET gene cause constitutive activation (without control) of its tyrosine–kinase activity and trigger malignant transformation. Genetic alterations of MET account for 3-5% of all cancers (a phenomenon called “addiction”) [1,21,22,23]. Yet, MET is over-expressed (excess production) in 90% of cancers, facilitating metastatic dissemination (a phenomenon known as “expedience”) [1,23,24]. Some tumors may produce HGF themselves or stimulate surrounding stromal cells to produce HGF, leading to autocrine or paracrine activation of MET. This creates a self-sustaining loop that encourages tumor growth and progression [25]. In particular, aberrant MET signaling can lead to increased cell motility, invasion, and disruption of normal tissue architecture, hallmarks of malignant progression. MET can also be activated through cross-talk with other receptors, even in the absence of its own ligand [26]. This can occur through heterodimerization with other receptor tyrosine kinases, which can amplify signaling pathways associated with tumorigenesis. The identification of MET as a cancer biomarker has played a significant role in the development of therapies in oncology such as MET tyrosine kinase inhibitors (TKIs), antibodies, and antibody–drug conjugates (ADCs) designed to target MET.

## 2. MET Structure

**Figure 1:**
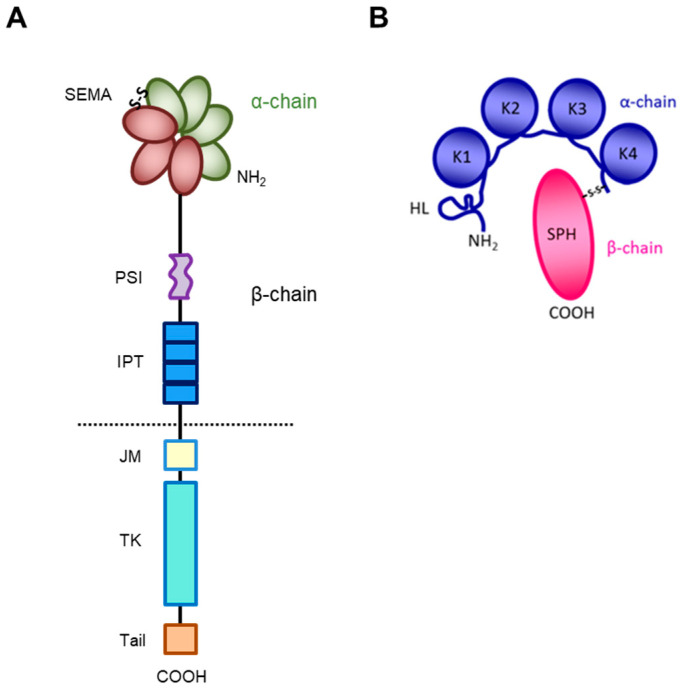
Structure of HGF/MET couple. (**A**) MET tyrosine kinase receptor is formed by α and β chains, which together constitute the semaphorin (SEMA) domain involved in HGF ligand binding. Extracellularly, the MET β-chain is also composed of a plexin–semaphorin–integrin (PSI) domain and four immunoglobulin-like plexin transcription factors (IPT) domains. Intracellularly, it contains a regulatory juxtamembrane (JM) domain, a tyrosine kinase (TK) domain, and a C-terminal tail. (**B**) HGF is also formed by α and β chains. The α-chain of the HGF ligand is constituted by an N-terminal harpin loop (HL) and four kringle domains (K1-K4). Instead, the β-chain is composed of a serine protease homology domain (SPH) which lacks proteolytic activity.

The receptor encoded by the MET gene is a dimeric protein proteolytically processed and glycosylated from a precursor of 170kDa (Figure 1A): The short α-chain (50kDa) is exposed to the surface of the cell and is covalently bound through disulfide bridges to the long transmembrane β-chain (145kDa) consisting of (i) an extracellular domain forming the functional domain called SEMA (Semaphorin), containing the binding site for the HGF factor (Figure 1B), with the α-chain [27,28,29,30]; (ii) a plexin–semaphorin–integrin (PSI) homology domain endowed with disulfide exchange isomerase activity [31]; and (iii) four IPT (immunoglobulin-like, plexins, transcription factors) domains, two of which (IPT3 and 4) contain a second high-affinity site for HGF binding [32].

**Figure 2:**
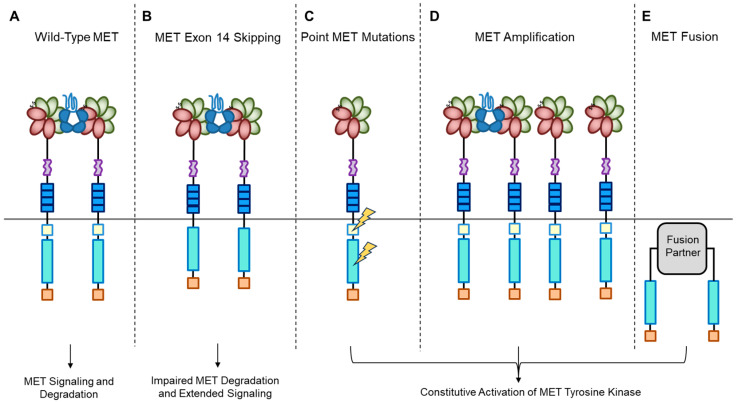
MET oncogenic alterations leading to receptor and downstream signaling activation.

A short transmembrane segment joins the extracellular to the intracellular portion that contains the functional domain endowed with tyrosine kinase activity [10]. MET/HGF interaction unleashes the kinase activity and the C-terminal tail of the receptor is phosphorylated, followed by signal transduction and MET degradation to terminate the signal (Figure 2A) [10,33]. Genetic alterations responsible for uncontrolled, constitutive activation of MET (Figure 2B–E) lead to abnormal cell growth (neoplastic transformation) and migration into tissues in an uncontrolled manner (invasion and metastasis) [34,35], featuring the invasive growth phenotype.

## 3. MET Cross-Talk with Other RTKs

The MET receptor can interact with other RTKs, among which are epidermal growth factor receptor (EGFR), human epidermal growth factors (HERs) 2 and 3 (also known as ERBB), and rearranged during transfection (RET) receptor [26]. The interaction between MET and EGFR is particularly important in non-small-cell lung carcinoma (NSCLC). EGFR signaling can lead to MET phosphorylation, which, especially when combined with the presence of ERBB3, can significantly increase the activity of these receptors [36]. Inhibition of EGFR or MAPK reduces MET activation and protein levels, highlighting the potential of combination therapy targeting EGFR and MET in NSCLC [36]. This relationship helps cancer cells survive and grow, and can also contribute to them being resistant to drugs. Some studies have shown that lung cancer cells resistant to gefitinib or erlotinib often have an increase in MET (MET amplification) [37,38,39]. MET amplification leads to ERBB3-dependent PI3K activation, traditionally associated with the EGFR/ERBB family. This indicates that MET’s role in resistance might extend beyond individual receptors, potentially affecting a range of ERBB-driven cancers, and this highlights the need for targeting this pathway in combinational treatment strategies [37]. A preference for HER3 among EGFR-family RTKs for MET-dependent tyrosine phosphorylation was observed in multiple MET-amplified cancer cell lines [40]. MET amplification is a consistent mechanism of acquired resistance in a number of other oncogene-driven molecular subsets of NSCLC post-tyrosine kinase inhibition [41]. A recent study conducted by Salokas et al. [42] revealed important interactions between MET and other receptors, such as the neurotrophic receptor tyrosine kinase 3 (NTRK3), platelet-derived growth factor receptor β (PDGFRβ), insulin receptor (INSR), and tyrosine protein kinase receptor (TYRO3). These unexpected MET interactions contribute to our understanding of the various cellular processes and signaling networks with key roles in enhanced cancer cell motility, invasion, and metastatic potential. Collective efforts to identify additional networks of MET interactions are crucial to enhancing our comprehension of oncogene signaling pathways and to develop new therapeutic strategies.

## 4. MET Exon 14 Skipping

**Figure 3:**
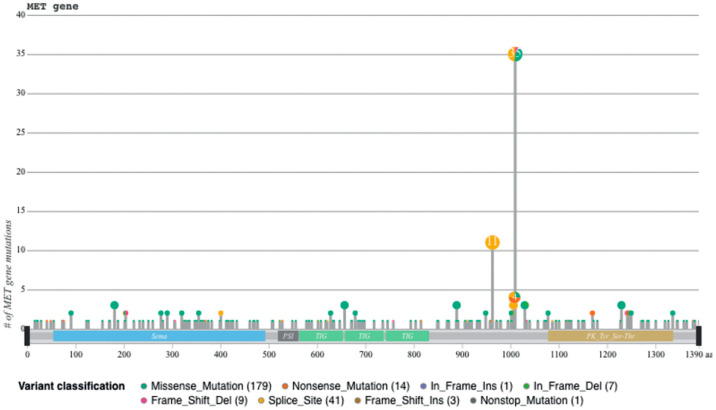
Sequences flanking exon 14 are mutational hotspots, from [45].

Recently, we developed a bioinformatic tool to create an auto-updatable catalog (“MET observatory”) of the MET genetic alterations in cancer [43]. The catalog of genetic alterations results from the following data collection databases: The Cancer Genome Atlas (TCGA), Catalogue of Somatic Mutations in Cancer (COSMIC), and ClinVar datasets. The MET “observatory” revealed a peculiar mutational distribution. The most frequent lesions are not mutations affecting the tyrosine kinase domain (as in the case of similar oncogenes), but the sequences flanking exon 14 (Figure 2 and Figure 3) [44]. The point mutations occur in the splicing acceptor or donor sites, resulting in the “skipping” of the whole exon 14 (METΔ14). This exon encodes a protein tract immediately below the plasma membrane (JM, Figure 1A and Figure 2B). We deeply investigated this hotspot mutation and showed that the METΔ14 activation is ligand-dependent [45]. A lack of JM leads to receptor activation, exacerbating the invasive growth phenotype [46]. There are a number of considerations in the finding of MET exon 14 skipping mutation(s): (i) It represents a targetable alteration in cancer, particularly in NSCLC [44,47,48]; (ii) it occurs in 2-4% of NSCLC and, with less frequency, in gastrointestinal carcinomas, gliomas, sarcomas, and cancers of unknown primary origin (CUPs) [48,49,50]; (iii) drugs like crizotinib, capmatinib, ensartinib, and tepotinib have shown promising results in clinical trials for NSCLC patients with this mutation [51,52,53,54,55,56,57,58]; (iv) research on MET exon 14 skipping is ongoing, and new therapies are continually being developed and tested; and (v) while targeted therapies against MET, particularly METΔ14, have shown promise, resistance to these drugs can develop over time [59].

## 5. MET Addiction

MET mutations affecting its catalytic or regulatory sites are sporadic, but they exist. The first activating mutations were identified in hereditary papillary renal carcinoma (HPRC), suggesting their causal role in this tumor [19]. Similar mutations were found in sporadic renal carcinoma, inducing constitutive kinase activation and oncogene “addiction” (Figure 2C), meaning that cancer cells rely heavily on a single hyperactive oncogene for their growth and survival. Transgenic mouse experiments confirmed the oncogenic potential of these mutations [60]. Activating mutations were later identified in hepatocellular carcinoma, head and neck cancers, oropharynx squamous cell cancer, gastric cancer, CUPs, and colorectal cancer [1,61]. Another commonly observed MET alteration occurs through gene amplification, with a prevalence rate of 3–5% across tumors [62,63,64,65,66,67,68,69]. Tumor cells become addicted to MET, justifying the use of targeted therapies (small molecules or antibodies [1]. MET targeting in cancer has proven its efficiency both in preclinical models and in patients [62,63,64,65,66,67,68,69,70]. MET gene amplification results in an increased number of MET receptors at the cell surface, which leads to constitutive kinase activation [22]. MET gene amplification can make cells independent of, or hypersensitive to, ligand stimulation (Figure 2D), enhancing MET signaling and driving cancer growth [63,71,72,73,74,75]. It is essential to consider MET amplification against the backdrop of widespread chromosomal aberrations seen in cancers, including the prevalent condition of cellular aneuploidy. Trisomy of chromosome 7 is a common occurrence in various cancers and can act as a pan-cancer genetic marker, potentially confounding the assessment of MET amplification. Unlike chromosome 7 trisomy, MET gene amplification is specifically selected during cancer development and functions as a cancer driver [76].

A recent study indicated that possessing at least five copies of the MET gene leads to a dependency on its signaling, thereby providing a rationale for targeted therapies [77]. While a specific threshold has not been universally agreed upon in clinical settings, accurate patient stratification for MET-directed treatments is critical. Techniques such as fluorescence in situ hybridization (FISH) can differentiate true MET gene amplification from chromosome 7 polysomy. In cases of polysomy, the ratio between the MET gene and the centromere of chromosome 7 (MET/CEN7) remains unchanged; however, an elevated ratio indicates true amplification of the MET gene. This distinction is essential for identifying patients who are most likely to respond to MET-targeted therapies [78]. Recent advancements have enabled next-generation sequencing (NGS) to establish criteria to differentiate between MET gene amplification and chromosome 7 polysomy in cancer, showing a high concordance with FISH analysis [79]. However, the performance of NGS in detecting polysomy in plasma samples is not as high as in tissue samples, indicating that there is room for improvement with non-invasive testing methods.

When MET amplification occurs in cancer cells already treated with targeted therapies, it can lead to treatment resistance [37,80]. This resistance driven by MET amplification highlights the adaptability of cancer cells. While targeted therapies designed to inhibit specific pathways can be highly effective initially, cancer cells often find ways to by-pass these interventions, making long-term treatment success challenging. One potential strategy to address resistance related to MET amplification is the development of combination therapies. Combining MET inhibitors with other targeted agents or immunotherapy may help overcome resistance by targeting multiple pathways simultaneously [81]. Biomarker testing for MET amplification is crucial for identifying patients who may benefit from MET-targeted therapies or combination treatments [81]. This underscores the importance of personalized medicine in cancer care.

## 6. The “Flare Effect”

When MET targeted therapies suffer from drug resistance, the line of treatment is discontinued. Previous studies have shown that withdrawal of MET tyrosine kinase receptor inhibition leads to a post-treatment increase in cancer cell proliferation due to a transient hyper-phosphorylation phase, which culminates in the “MET burst” [82], i.e., the “flare effect”. The molecular mechanisms behind this effect remain unclear, but are critically important for patients. Recently, our laboratory identified a positive feedback loop mediated by the AKT/mTOR pathway that leads to the “MET burst” after treatment withdrawal [83]. This feedback loop enhances MET translation through activation of p70S6K and 4EBP1 and causes MET hyper-phosphorylation via inactivation of the tyrosine–phosphatase PTP1B. These data suggest that the use of mTOR inhibitors during MET-targeted therapy may prevent the occurrence of the “flare effect”.

## 7. MET Fusion

Previously, TPR-MET was the only recognized MET gene rearrangement in human tumors predominantly found in gastric cancers. However, extensive analyses of the TCGA tumor database have revealed new hybrid proteins, where the intracellular domain of MET, or even the full-length MET, is fused with various partners (Figure 2E) [84]. These fusion events can result in novel hybrid proteins with altered functional properties and are a significant aspect of the genetic alterations observed in certain cancers. Some of these partners include proteins with a dimerization “coiled-coil” (CC) motif known for promoting dimerization, such as C8orf34, BAIAP2L1, TFG, or KIF5B. These fusions instigate ligand-independent dimerization of MET, causing continuous kinase activity that can lead to tumor formation. While these fusions are infrequent, they have been detected in lung adenocarcinomas, hepatocellular carcinomas, papillary renal carcinomas, and thyroid carcinomas [84].

Another notable gene rearrangement is between MET and PTPRZ1, a gene encoding a tyrosine phosphatase, prevalent in certain brain tumors like low-grade gliomas and pediatric glioblastomas [85]. The full-length MET coding sequence is present in the PTPRZ-MET fusion transcript, and the MET protein is overexpressed and endowed with enhanced kinase activity [86]. The exact mechanism of increased MET expression in tumors with PTPRZ-MET genes fusion is not fully understood. Yet, there is evidence suggesting that tumors harboring these gene fusions can be responsive to anti-MET monotherapy, as seen with PTPRZ1-MET in pediatric gliomas [87] and KIF5B-MET in lung cancers [88]. MET gene fusions also occur in melanomas, involving various N-terminal partners fused with the intracellular MET domain [89].

Different MET genetic alterations can induce either HGF-dependent or ligand-independent activation of the kinase, with a shared characteristic of driving invasive growth. Tumor cells become addicted to MET, making them susceptible to targeted therapies.

## 8. MET Oncogene Expedience

In multiple tumor types, the activation of MET is a subsequent event that intensifies the malignant characteristics of cells that have already undergone transformation. In such instances, the abnormal activation of MET may result from transcriptional upregulation induced by other oncogenes; environmental factors such as hypoxia or radiation [90,91]; or substances produced by the reactive stroma, such as inflammatory cytokines, pro-angiogenic factors [92,93], and even HGF itself [94,95]. Hypoxia is one condition that induces MET transcription [90]. This microenvironmental regulation of MET expression might explain why anti-angiogenic therapy leads to MET overexpression, constitutive kinase activation, MET-dependent invasive growth, and distant metastases [96,97,98]. Accordingly, concurrent MET and VEGF targeting mitigates tumor aggressiveness in pancreatic carcinoma [99]. When receptors are increased, they can cluster to form receptor oligomers. This interaction can trigger mutual activation, making cells sensitive to ligand concentrations that would normally be under the threshold to elicit a response. Unlike the concept of “oncogene addiction,” where the oncogene is a primary driver, the inappropriate activation of MET leading to “oncogene expedience” is a result, rather than the cause, of the transformed phenotype, and may facilitate the cancer’s progression to metastatic spreading. Therefore, alterations impacting the MET promoter merit attention. Re-analysis of the TCGA dataset may uncover a notable decrease in MET promoter methylation in cancer patients, indicating transcription activation and increased expression of MET [26]. This observation underscores the need to further understand central regulatory mechanisms. Gene expression is not solely governed at the transcription level; post-transcriptional control mechanisms affecting translation efficiency and messenger RNA stability may play crucial roles in observed cancer deregulations.

## 9. Clinical Impact of MET Oncogene

A clinical study of a large number of solid tumors tested at the same cancer center revealed MET amplification in 2.5% of 1115 patients with advanced cancers [100]. The prevalence was highest in renal cell carcinoma (RCC, 14%) followed by adrenocortical tumors (15%), gastroesophageal (6%), breast (5%), and ovarian cancers (4%) [100]. This is in agreement with previous works concentrated on a single tumor type [71,101,102,103,104,105,106]. MET-amplified tumors were associated with a higher histologic grade and development of more metastatic sites [100]. Approximately 2% of glioblastomas [107] and 12% of melanomas [75] exhibited MET amplification according to whole-genome analysis. Approximately 3% of advanced NSCLC cases harbor point mutations or deletions in MET exon 14 or its flanking introns [47], while around 1% of poor survival NSCLC patients are associated with de novo MET amplification [108]. Resistance to EGFR TKIs in lung cancer patients is commonly determined by MET amplification. This was reported in 5% of patients after first-generation EGFR TKIs and 10% of osimertinib (mutant T790M-selective EGFR TKI)-treated NSCLC patients [109,110]. Recently, MET amplification has also been detected in ALK-rearranged NSCLC patients treated with ALK TKI [111]. In the absence of MET amplification, MET has been found to be overexpressed in a variety of cancers, such as RCC [19], NSCLC [112], malignant pleural mesothelioma (MPM) [113], glioblastoma multiforme (GBM) [114], and gastric cancer (GC) [115]. High levels of MET expression are associated with poor survival outcomes in patients with gastrointestinal malignancies [116]. Numerous meta-analyses have demonstrated that abnormal activation of the MET pathway in cancerous tissue, characterized by overexpression of the MET gene, gene amplification, exon 14 skipping, and other activating mutations, consistently correlates with decreased survival and adverse outcomes (reviewed in [117]). These analyses primarily focus on NSCLC, as well as cancers of the breast, head and neck, colorectum, stomach, pancreas, and other parts of the gastrointestinal tract. The analyses of patient data in these studies have underscored a clear correlation between MET expression or mutation and survival rates. Patients exhibiting MET amplification or overexpression, indicative of heightened MET pathway activity, have consistently shown poorer prognoses across various cancer types. This trend is particularly pronounced in cancers with the highest prevalence of MET amplification, such as renal cell carcinoma and adrenocortical tumors. In NSCLC, MET exon 14 mutations or amplifications are significant predictors of reduced survival, reflecting the aggressive nature of MET-driven cancer phenotypes. Moreover, multiple studies have highlighted the usefulness of MET biomarkers for pinpointing patients who benefit most from targeted HGF/MET therapies, whether used alone or in combination [44,51,55,118,119]. The most significant predictive value for these biomarkers has been noted in responses to savolitinib in renal cancer [101] and tepotinib in NSCLC [51,55]. However, certain research, particularly that emphasizing MET expression, has not proven to be very useful in these categorizations, possibly due to a lack of standardized methods, especially in immunohistochemistry scoring systems, or because the cancer cells are not dependent on the MET pathway despite the presence of overexpression. Assessments based on amplification and mutation tend to be less affected by these issues.

In the last decade, several MET inhibitors, including monoclonal antibodies [120,121,122,123,124,125] and small molecules such as TKI [51,52,53,54,55,56,57,58,70,102,112,126,127,128,129,130], have been developed and tested in clinics. The lack of functional molecular stratification in patients with genetically susceptible tumors, such as MET∆14, has led to the failure of some trials. These studies have indicated the necessity of genetic assessment to identify oncogene-addicted tumors, thereby enriching the pool of patients who respond to treatment. In 2020, Japan and the USA approved two MET TKIs, tepotinib and capmatinib, respectively, for the treatment of advanced NSCLC with the MET exon 14 skipping mutation. As discussed above, MET inhibitors might be beneficial when used in combination with other targeted therapies to block HGF-dependent survival function [1,61,131,132,133]. Furthermore, other innovative therapeutic techniques, such as ADCs combining the specificity of antibodies with the potency of cytotoxic agents [134,135,136], have been tested. These therapeutic agents could be very functional in patients overexpressing the MET receptor.

## 10. Conclusions

In summary, since its discovery as an oncogene, MET has been found to undergo mutations, amplifications, or rearrangements in a wide range of cancers, spanning from the early stages of tumor initiation to instances of therapeutic resistance and recurrence. The analysis of genetic changes occurring within the MET receptor has revealed a distinct pattern of mutations, primarily affecting the sequences flanking exon 14. In clinical practice, the presence of MET genetic abnormalities is a critical factor in identifying tumors that rely on this oncogene (MET oncogene-addiction), making such patients potential candidates for targeted therapies. Where MET is genetically altered and is the first cause of transformation, pharmacological inactivation may result in complete cancer remission.

In physiological settings, fine-tuning the quantity of MET receptors is essential for wound healing and tissue regeneration. This “rescue” function is usurped by neoplastic cells to foster cell survival and facilitate invasion and the spread of metastases. Thus, wild-type MET can function as an “oncogene expedient”, enhancing the impact of other oncogenes and promoting malignant progression. Even if a tumor lacks MET mutations, it may still respond to treatments that target MET or its downstream pathways. Such targeted interventions may reduce the survival of the primary tumor, as well as its invasion and metastatic spread, thus impeding cancer progression.

## Data Availability

Data is contained within the article.

## Abbreviations

ADC, antibody–drug conjugate; CC, coiled-coil; CUP, cancer of unknown primary origin; COSMIC, Catalogue of Somatic Mutations in Cancer; ECM, extracellular matrix; FISH, fluorescence in situ hybridization; EGFR, epidermal growth factor receptor; GBM, glioblastoma multiforme; GC, gastric cancer; Grb2, growth factor receptor-bound protein 2; HGF, hepatocyte growth factor; HER, human epidermal growth factor; HL, harpin loop; HPRC, hereditary papillary renal carcinoma; INSR, insulin receptor; IPT, immunoglobulin-like, plexin transcription factors; JM, juxtamembrane domain; MPM, malignant pleural mesothelioma; NGS, next-generation sequencing; NTRK3, neurotrophic receptor tyrosine kinase 3; NSCLC, non-small-cell lung cancer; PDGFRβ, platelet-derived growth factor receptor β; PI3K, phosphoinositide 3-kinase; PLCγ, phospholipase Cγ; PSI, plexin–semaphorin–integrin; RET, rearranged during transfection; RCC, renal cell carcinoma; RTK, receptor tyrosine kinase; SEMA, semaphorin; SF, scatter factor; SPH, serine protease homology; STAT3, signal transducer and activator of transcription 3; TCGA, The Cancer Genome Atlas; TKI, tyrosine kinase inhibitor; TPR, translocated promoter region; TYRO3, tyrosine protein kinase receptor.
